# Improved motor and cognitive performance with sodium nitrite supplementation is related to small metabolite signatures: a pilot trial in middle-aged and older adults

**DOI:** 10.18632/aging.100842

**Published:** 2015-11-30

**Authors:** Jamie N. Justice, Lawrence C. Johnson, Allison E. DeVan, Charmion Cruickshank-Quinn, Nichole Reisdorph, Candace J. Bassett, Trent D. Evans, Forrest A. Brooks, Nathan S. Bryan, Michel B. Chonchol, Tony Giordano, Matthew B. McQueen, Douglas R. Seals

**Affiliations:** ^1^ Department of Integrative Physiology, University of Colorado Boulder, Boulder, CO 80309, USA; ^2^ Integrated Department of Immunology, University of Colorado Anschutz Medical Campus and National Jewish Hospital, Denver, CO 80045, USA; ^3^ Baylor College of Medicine, Houston, TX 77030, USA; ^4^ Division of Renal Diseases & Hypertension, University of Colorado Denver, Aurora, CO 80045, USA; ^5^ TheraVasc Inc., Cleveland, OH 44106, USA

**Keywords:** aging, metabolomics, neuromuscular, precision medicine, nitrates/nitrites

## Abstract

Advancing age is associated with reductions in nitric oxide bioavailability and changes in metabolic activity, which are implicated in declines in motor and cognitive function. In preclinical models, sodium nitrite supplementation (SN) increases plasma nitrite and improves motor function, whereas other nitric oxide-boosting agents improve cognitive function. This pilot study was designed to translate these findings to middle-aged and older (MA/O) humans to provide proof-of-concept support for larger trials. SN (10 weeks, 80 or 160 mg/day capsules, TheraVasc, Inc.) acutely and chronically increased plasma nitrite and improved performance on measures of motor and cognitive outcomes (all p<0.05 or better) in healthy MA/O adults (62 ± 7 years). Untargeted metabolomics analysis revealed that SN significantly altered 33 (160 mg/day) to 45 (80 mg/day) different metabolites, 13 of which were related to changes in functional outcomes; baseline concentrations of 99 different metabolites predicted functional improvements with SN. This pilot study provides the first evidence that SN improves aspects of motor and cognitive function in healthy MA/O adults, and that these improvements are associated with, and predicted by, the plasma metabolome. Our findings provide the necessary support for larger clinical trials on this promising pharmacological strategy for preserving physiological function with aging.

## INTRODUCTION

Advancing age is associated with declines in several domains of motor function including muscle strength/power, endurance, balance, dexterity and locomotor performance [1-3]. These changes can lead to functional limitations and increased risk of disability, falls, and loss of independence [4-7]. Given the marked increases in the number of older adults expected in the coming decades, establishing the efficacy of interventions that enhance motor function in the late middle-aged and older (MA/O) population is a high biomedical research priority [8-10].

There is strong evidence that regular exercise is an effective strategy for optimizing physical function with aging [11, 12], however, physical activity decreases with age and most MA/O adults fall well short of the recommended weekly guidelines [13-15]. As such, there is growing interest in pharmacological compounds, including dietary supplements and nutra-ceuticals, that may exert at least some of the benefits of physical activity and other healthy lifestyle practices on physical function with aging [8].

Recently, the potential health benefits of nitrate and nitrite supplementation have received considerable attention. Nitrate and nitrite are precursors of nitric oxide (NO), a ubiquitous gaseous signaling molecule that plays a critical role in systemic physiological function. Studies assessing the effects of dietary and/or pharmacological supplementation with nitrates or nitrites on motor function in healthy young adults have yielded mixed results [16-20]. However, NO bioavailability declines with aging and, therefore, NO-boosting treatments may have greater effects on physiological function in MA/O adults. Consistent with this possibility, we recently showed that supplementation with sodium nitrite in the drinking water for 8 weeks improved grip strength, locomotor activity and endurance performance in old mice [21]. Presently, it is unknown if sodium nitrite supplementation can improve motor function in MA/O humans.

To obtain preliminary support for this possibility, we performed a small-scale pilot (feasibility) trial to assess the potential efficacy of sodium nitrite supplementation for improving multiple domains of motor function in healthy MA/O adults. Two different doses of sodium nitrite (80 and 160 mg/day, TheraVasc, Inc.) were assessed over a 10-week treatment period using a randomized, placebo controlled, double-blind design. Because cognitive function is closely linked to motor performance [22, 23], and treatment with sodium nitrite or NO donors improves learning and memory in rodents [24], we took the opportunity to also assess effects on two simple, time-efficient measures of processing speed and executive function. Finally, to gain initial insight into the molecular signaling mechanisms underlying any improvements in motor or cognitive function observed, we assessed the plasma metabolome before and after sodium nitrite supplementation using an untargeted approach. In an extensive post-hoc analysis, we determined changes in small metabolite signatures with treatment, the relation between these changes and improvements in function, and identified plasma metabolites at baseline that predicted responsiveness to treatment.

## RESULTS

### Subject characteristics and safety

Characteristics of the groups are shown in Table 1. At baseline and following 10 weeks supplementation with 80 mg or 160 mg sodium nitrite per day or placebo, no group differences in body composition characteristics (total body mass, lean body mass and regional lean mass), basic blood panel (fasting glucose, insulin and cholesterol) or habitual daily activity were observed (p>0.05, all). Sodium nitrite was well tolerated. No severe adverse events occurred in any group (for details, see [25]). Methemoglobin did not exceed peak levels of 1.5% in any group (12% safety cut-off). Two subjects were withdrawn due to moderate adverse events (placebo: dizziness; high dose: headache).

**Table 1:** Subject characteristics before and after 10 weeks of placebo and sodium nitrite supplementation

	Placebo		Nitrite (80 mg/day)		Nitrite (160 mg/day)	
	Baseline	Week 10	Baseline	Week 10	Baseline	Week 10
n (women)		10 (4)		10 (5)		10(5)
Age (years)	62 ± 8	--	60 ± 6	--	64 ± 6	--
BMI (kg/m^2^)	25.9 ± 2.7	25.8 ± 2.8	24.5 ± 3.2	24.6 ± 2.8	24.2 ± 3.0	24.4 ± 3.2
Body mass (kg)	75.1 ± 10.2	74.8 ± 10.1	70.6 ± 9.3	71.3 ± 9.2	70.4 ± 10.2	70.9 ± 10.1
Lean mass (kg)	48.6 ± 14.3	50.1 ± 11.8	47.2 ± 7.7	47.9 ± 8.4	47.6 ± 8.2	48.2 ± 8.4
Body fat (%)	30.4 ± 11.1	30.0 ± 11.3	29.7 ± 7.9	29.4 ± 8.7	27.9 ± 10.0	28.1 ± 10.3
Fasting glucose (mg/dL)	94 ± 5	94 ± 6	90 ± 6	90 ± 5	93 ± 4	91 ± 5
Fasting insulin (μU/mL)	8 ± 2	10 ± 3	9 ± 3	8 ± 2	8 ± 3	7 ± 3
Total cholesterol (mg/dL)	164 ± 22	158 ± 22	175 ± 25	174 ± 28	180 ± 19	183 ± 25
HDL-cholesterol (mg/dL)	59 ± 6	58 ± 6	58 ± 4	60 ± 4	57 ± 4	59 ± 4
LDL-cholesterol (mg/dL)	90 ± 5	87 ± 4	100 ± 6	101 ± 7	109 ± 6	108 ± 8
Triglycerides (mg/dL)	79 ± 8	67 ± 5	70 ± 6	70 ± 13	75 ± 6	80 ± 9
Activity (steps/wk)	60.7 ± 24.3	55.4 ± 18.3	70.2 ± 36.4	67.4 ± 35.5	67.7 ± 32.0	66.0 ± 36.2

Data are mean ± SD. HDL, high-density lipoprotein; LDL, low-density lipoprotein. Activity is reported as value × 10,000 steps.

### Plasma nitrite

Plasma nitrite concentrations did not differ among groups at baseline (Table 2). Levels increased 6- to 25-fold at 30 min after acute ingestion of the initial dose of sodium nitrite in the low- (80 mg/day) and high- (160 mg/day) dose sodium nitrite groups, respectively, with no change observed in the placebo group. These observations demonstrate that the treatment acutely increased plasma nitrite concentrations in our subjects.

**Table 2:** Subject characteristics before and after 10 weeks of placebo and sodium nitrite supplementation

	Placebo		Nitrite (80 mg/day)		Nitrite (160 mg/day)	
	Baseline	Week 10	Baseline	Week 10	Baseline	Week 10
**Acute (baseline and 30 min after 1^st^ dose)**						
**Nitrite**	0.23 ± 0.24	0.20 ± 0.19	0.47 ± 0.47	3.34 ± 1.88*	0.27 ± 0.31	7.13 ± 3.36*
**Chronic (baseline and 12-18 h after final dose at week 10)**						
**Nitrite**	0.20 ± 0.21	0.26 ± 0.16	0.21 ± 0.17	0.47 ± 0.27^†^	0.15 ± 0.15	0.37 ± 0.21^†^

* p<0.05;

† p<0.1

Following 10 weeks of sodium nitrite supplementation, a trend was observed for a small elevation of plasma nitrite levels 12-18 h after ingestion of the last capsule in 80 mg/day and 160 mg/day nitrite groups (0.05<p<0.1; Table 2). Consistent with its short half-life, these data suggest that the sodium nitrite supplementation produced a small chronic increase in circulating nitrite con-centrations [26].

### Sodium nitrite supplementation and motor function

#### Rate of torque development and muscle strength

**Figure 1:**
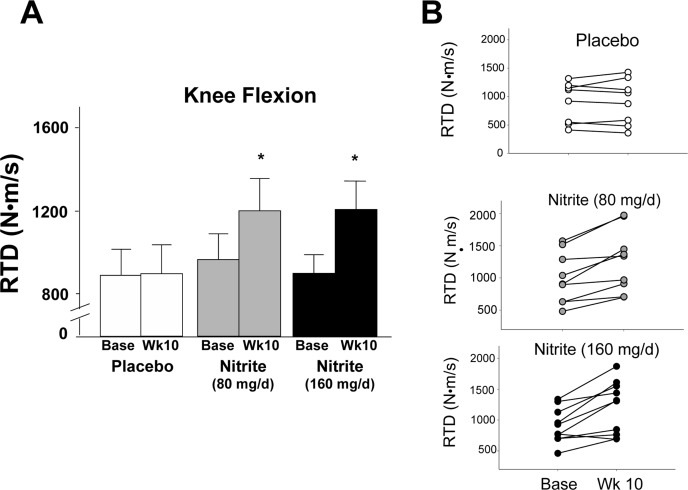
Rate of torque development (RTD) for the knee flexors at baseline and following 10 weeks of placebo (white) or sodium nitrite supplementation (gray – 80 mg/day; black – 160 mg/day). Sodium nitrite supplementation improved knee flexor RTD as shown by time × treatment interaction for three groups **(A);** * p<0.05). Individual subject data **(B)** indicated that knee flexor RTD changed little across 10 weeks for the 10 subjects in the placebo group (white circles), but improved for most subjects taking sodium nitrite 80 mg/day (gray circles), and subjects supplemented with 160 mg/day (black circles).

**Figure 2:**
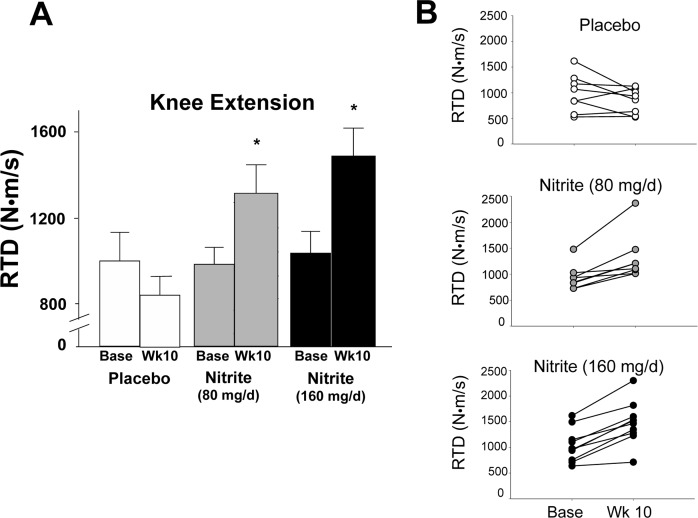
Rate of torque development (RTD) for the knee extensors at baseline and following 10 weeks of placebo (white) or sodium nitrite supplementation (gray – 80 mg/day; black – 160 mg/day). Sodium nitrite supplementation improved knee extensor RTD as shown by time × treatment interaction for three groups **(A);** * p<0.05). Individual subject data **(B)** indicated that knee extensor RTD changed little across 10 weeks for placebo (white circles), but improved for most subjects taking sodium nitrite 80 mg/day (gray circles), and subjects supplemented with 160 mg/day (black circles).

The motor outcomes demonstrating the most consistent improvements across groups (panel A) and individual participants (panel B) were knee flexor and extensor rate of torque development (RTD) (Figures 1 and 2). Specifically, knee flexor and knee extensor RTD were improved by 33 and 43% and 26 and 34%, respectively, with 80 mg/day and 160 mg/day sodium nitrite compared with placebo (p<0.01); the changes were not different between the two doses (p>0.05 between 80 and 160 mg/day). For the knee flexors, 7 of 10 individuals in the 80 mg/day group and 8 of 10 individuals in the 160 mg/day nitrite group demonstrated ≥10% improvements in RTD following sodium nitrite supplementation (Figure 1B). For the knee extensors, improvements of ≥10% were observed in 8 of 10 subjects in both the low- and high-dose groups (Figure 2B). The improvements in RTD were observed in the absence of significant changes in maximum voluntary contraction (MVC) torque for knee extensors or flexors (p>0.05, Table 3). Sodium nitrite supplementation did not alter grip strength, although a trend for improvement was observed in the 80 mg/day nitrite group (p<0.1, Table 3).

**Table 3:** Measures of motor function before and after 10 weeks of placebo and sodium nitrite supplementation

	Placebo		Nitrite (80 mg/day)		Nitrite (160 mg/day)	
	Baseline	Week 10	Baseline	Week 10	Baseline	Week 10
**Balance**						
Rapid step errors	3.2 ± 1.4	3.3 ± 1.0	2.9 ± 1.7	2.0 ± 1.3*	3.5 ± 1.7	2.4 ± 1.5*
Rapid step time (s)	40.2 ± 6.6	38.1 ± 6.2	40.4 ± 4.8	39.6 ± 4.0	41.3 ± 4.3	38.7 ± 4.0
**Fatigability**						
Heel-rise time (s)	64 ± 32	56 ± 21	88 ± 40	121± 56^†^	92 ± 53	113 ± 55
Fatigue (FSS score)	23.8 ± 9.6	21.9 ± 9.0	25.2 ± 11.3	22.1 ± 11.8	17.6 ± 8.1	15.8 ± 10.0
**Muscle Strength**						
Grip strength (kg)	38.4 ± 9.2	37.6 ± 9.2	38.3 ± 6.9	41.5 ± 9.1^†^	37.9 ± 7.7	39.3 ± 6.7
MVC, Knee extensors (N·m)	54 ± 19	62 ± 18	73 ± 33	78 ± 31	60 ± 18	65 ± 24
MVC, Knee flexors (N·m)	107 ± 27	107 ± 31	103 ± 29	107 ± 24	91.8 ± 27	103 ± 22
**Mobility**						
400 m (s)	196 ± 21	184 ± 24	206 ± 20	209 ± 18	199 ± 20	195 ± 20
Timed up & go (s)	5.3 ± 1.1	5.5 ± 1.5	6.2 ± 0.9	6.1 ± 0.5	5.9 ± 0.5	5.5 ± 0.4
**Manual Dexterity**	
Pegboard time (s)	67 ± 17	63 ± 14	68 ± 10	64 ± 11	65 ± 9	65 ± 8

* p<0.05;

† p<0.1. FSS, fatigue severity scale; MVC, maximal voluntary contraction.

#### Cardiorespiratory fitness and muscle endurance

**Figure 3:**
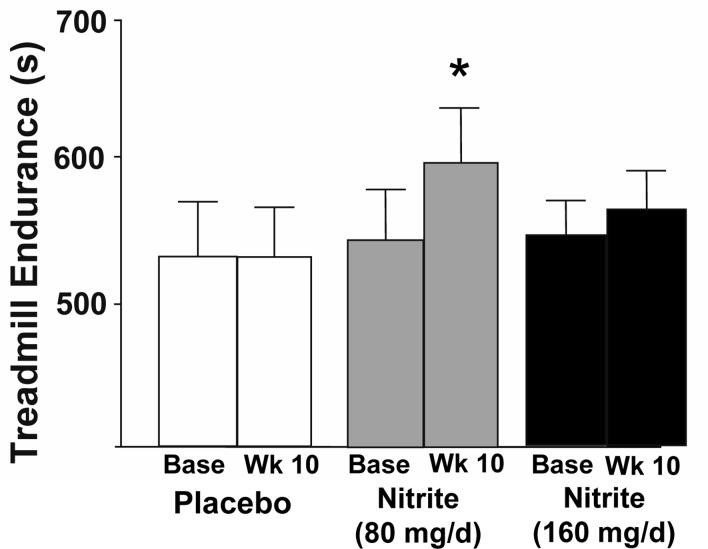
Endurance as measured by time to failure on a Balke treadmill task at baseline and following 10 weeks placebo (white) or sodium nitrite supplementation (gray – 80 mg/day; black – 160 mg/day). Sodium nitrite supplementation improved total duration subjects could sustain the treadmill test, especially at 80 mg/day supplementation (* p<0.05).

Compared with placebo, nitrite supplementation at the 80 mg/day dose improved maximal exercise capacity as assessed by treadmill time to exhaustion during the modified Balke incremental treadmill exercise protocol (Figure 3, p<0.05); no changes were observed for the 160 mg/day nitrite group. Heart rate, oxygen consumption (VO2max), ratings of perceived exertion, respiratory exchange ratio (index of hyperventilation) and minute ventilation during maximal exercise did not differ at baseline among groups nor change with nitrite supplementation (Table 4, p>0.05, all). No differences were observed at baseline among the groups or with treatment for any physiological response to submaximal levels of exercise during this treadmill protocol (data not reported).

**Table 4:** Responses to maximal aerobic exercise before and after 10 weeks of placebo and sodium nitrite supplementation

	Placebo		Nitrite (80 mg/day)		Nitrite (160 mg/day)	
	Baseline	Week 10	Baseline	Week 10	Baseline	Week 10
RPE	18 ± 0	19 ± 0	18 ± 0	19 ± 0	18 ± 1	18 ± 0
Heart rate (bpm)	166 ± 5	163 ± 5	169 ± 5	168 ± 5	163 ± 3	163 ± 3
RER	1.17 ± 0.03	1.16 ± 0.02	1.16 ± 0.03	1.17 ± 0.02	1.19 ± 0.02	1.12 ± 0.02
Ventilation (L/min)	68 ± 8	67 ± 7	67 ± 4	68 ± 5	65 ± 4	68 ± 4
VO_2_max (ml/(kg·min))	30.5 ± 6.9	30.0 ± 5.7	31.6 ± 6.7	31.8 ± 7.6	33.7 ± 9.2	33.6 ± 9.6

RPE, ratings of perceived exertion; RER, respiratory exchange ratio.

Time to failure for the heel-rise test was modestly improved with supplementation in the 80 mg/day group only (p<0.1, Table 3). There were no differences in fatigue (FSS score) at baseline or following treatment in any group (Table 3).

#### Balance, manual dexterity and mobility

The number of balance errors committed during the rapid step test were reduced by ∼30% after both doses of sodium nitrite treatment (p<0.05) in the absence of changes in rapid step time (p>0.1) (Table 3). The responders (i.e. > 10% improvement) for rapid step test errors were consistent with the responders for RTD. No changes were observed in pegboard times, 400-m walk time, or Timed Up & Go among groups (Table 3).

### Sodium nitrite supplementation and cognitive outcomes

**Figure 4:**
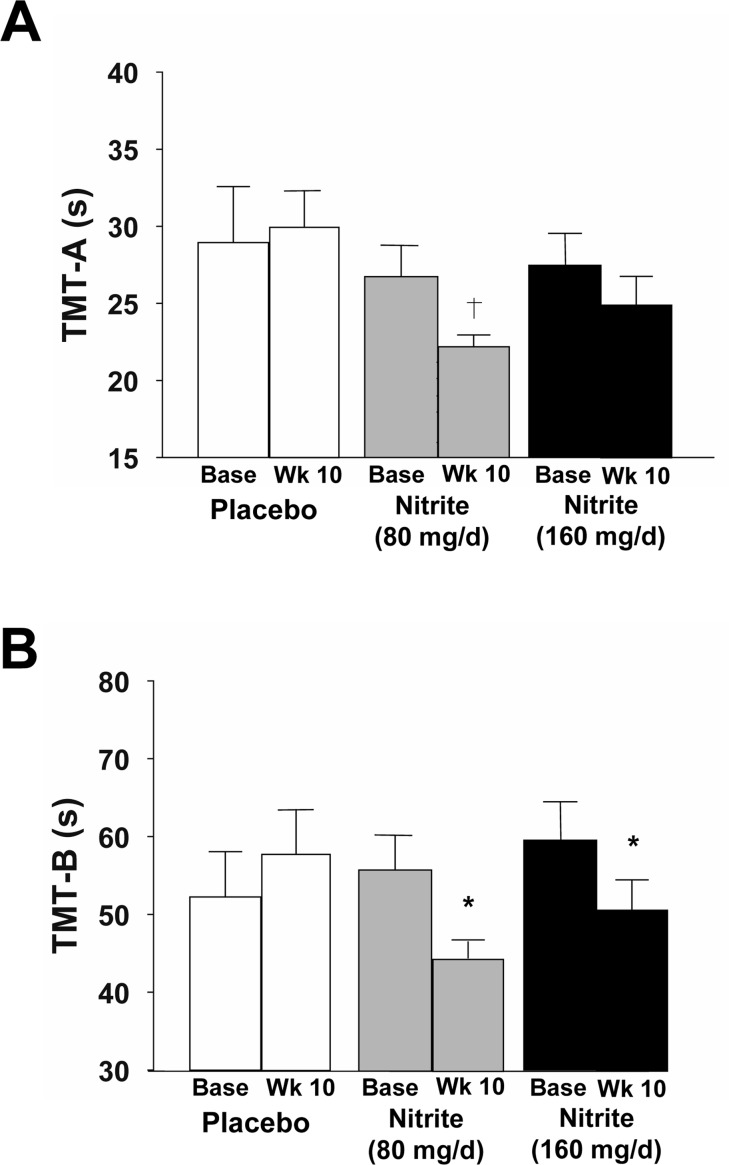
Cognitive function as assessed by time to complete the Trail Making Test-A (**A**, TMT-A) and -B(**B**, TMT-B) at baseline and following 10 weeks placebo (white) or sodium nitrite supplementation (gray - 80 mg/day; black - 160 mg/day). Sodium nitrite supplementation tended to improve time to complete TMT-A, an index of processing speed (**A;** † p<0.1). A significant time × treatment interaction was observed for TMT-B(**B;** * p<0.05), indicating that sodium nitrite supplementation improved an index of executive function.

Cognitive performance was improved with 10 weeks of sodium nitrite, as indicated by reductions in time to complete the Trail Making Tests B and A (TMT-B and TMT-A) (Figure 4). Time to complete TMT-B, which is an index of executive function, was improved by 18 and 14% in response to the high- and low-doses of sodium nitrite, respectively (Figure 4A, p<0.05). Of these, 15 responded consistently for TMT-B, RTD and step errors, though one of the 5 non-responders showed a 9% improvement and was just below the 10% responder criteria. A trend was observed for an improvement in processing speed (TMT-A) in response to the 80 mg/day of sodium nitrite (14% improvement, p<0.1, Figure 4B), but not 160 mg/day. Individual performance data are shown in Supplemental Figure 1.

### Sodium nitrite supplementation and the metabolome

#### Metabolomic signatures and sodium nitrite supplementation

In total, 3,063 metabolites were detected in the plasma of subjects in all groups and used in subsequent analyses of the plasma metabolome. We observed significantly altered metabolomic profiles following sodium nitrite supplementation compared to placebo. For some comparisons, the changes observed were specific for the 80 and 160 mg/day groups (Table 5). Of note, the changes in metabolite abundance occurred independent of any changes in subject characteristics or clinical blood markers. Overall, changes in 45 small molecules were observed in subjects administered 80 mg/day sodium nitrite (Supplemental Table 1) and 33 metabolites in subjects administered 160 mg/day (Supplemental Table 2). Both doses of sodium nitrite induced changes in abundance of compounds associated with glycerol-phospholipid, sphingolipid, carboxylic acid, peptide and pyridine metabolic pathways, whereas 80 mg/day of sodium nitrite also altered molecules involved in glycerolipid, fatty acyl and ribonucleoside metabolism.

**Table 5:** Metabolic pathways significantly altered by 10 weeks of sodium nitrite supplementation

Molecular Class	Number of Molecules Altered Within Each Pathway
**Nitrite (80 mg/day)**	
Glycerophospholipids	13
Sphingolipids	2
Glycerolipids	1
Fatty Acyls	3
Carbohydrates and Carbohydrate Conjugates	1
Carboxylic Acids and Derivatives	2
Imidazole Ribonucleosides and Ribonucleotides	1
Pyridines and Derivatives	1
Peptide Hormones	1
Unknown	20
**Nitrite (160 mg/day)**	
Glycerophospholipids	9
Sphingolipids	2
Carboxylic Acids and Derivatives	2
Polypeptide	1
Pyridines and Derivatives	1
Unknown	18

#### Relation of functional changes to the metabolome and clinical characteristics

To investigate the relation between alterations in small molecule concentrations and changes in motor and cognitive function, metabolites shown to change with sodium nitrite supplementation were tested for an association with a subset of functional outcomes that demonstrated the most consistent change following supplementation (noted by significant time × treatment interactions; RTD, rapid step errors, TMT-B, Table 6). In subjects receiving 80 mg/day sodium nitrite, increases in knee-extensor and knee-flexor RTD were significantly related to three metabolites each; two of these metabolites (C28 H44 O4 and C26 H58 N2 O2 S2) were related to the improvements in RTD for both muscle groups.

**Table 6:** Significant relations between specific metabolite concentrations and prioritized measures of physical and cognitive function following 10 weeks of sodium nitrite supplementation

	Beta	Standard Error	P-value
**Motor Outcomes**			
**Knee extensor rate of torque development**			
***Nitrite (80 mg/day)*** **Molecule**			
C28 H44 O4	−0.74	5.999	0.023
C26 H58 N2 O2 S2	0.75	4.474	0.02
C35 H62 N O15 P	−0.763	44.736	0.017
**Knee flexor rate of torque development**			
***Nitrite (80 mg/day)*** **Molecule**			
C28 H44 O4	−0.704	8.841	0.034
C26 H58 N2 O2 S2	0.752	7.155	0.019
LysoPE (18:3(9Z, 12Z, 15Z)/0:0)	0.785	7.028	0.012
***Nitrite (160 mg/day)*** **Molecule**			
C22 H23 F N6	0.692	9.275	0.039
PC(38:4)	−0.709	4.854	0.032
**Rapid Step Test**			
***Nitrite (80 mg/day)*** **Molecule**			
β-Methylcrotonyl coenzyme A	0.888	0.014	0.001
***Nitrite (160 mg/day)***			
C22 H23 F N6	0.647	0.042	0.043
C15 H43 N15 O2 S4	0.824	0.043	0.003
**Cognitive Outcome**			
**Trail Making Test B**			
***Nitrite (80 mg/day)*** **Molecule**			
LysoPC(18:1)	−0.768	0.621	0.009
5-Aminoimidazole-4-carboxamide-1-βD-ribofuranosyl- 5′-monophosphate	−0.792	1.235	0.006
***Nitrite (160 mg/day)*** **Molecule**			
C37 H58 N3 P3	0.649	0.789	0.042
C37 H59 N P4	−0.779	0.496	0.008

Improvements in the number of errors during the rapid step test were significantly related to changes in one metabolite, β-Methylcrotonyl coenzyme A, in the 80 mg/day group. Changes in TMT-B were significantly associated with changes in two known metabolites, a lysophosphatidylcholine (LysoPC 18:1) and a ribonucleoside (5-Aminoimidazole-4-carboxamide-1-βD-ribofuranosyl-5′-monophosphate). Several meta-bolites significantly altered by 160 mg/day sodium nitrite were related to improvements in knee-flexor RTD, rapid step errors, and TMT-B (Table 6). Importantly, the diverse profile of metabolites that were significantly associated with improvements in motor and cognitive function indicates the broad metabolic effects of sodium nitrite supplementation.

#### Baseline metabolomic predictors of functional responsiveness to treatment

Baseline metabolite concentrations were assessed for their ability to predict an individual's responsiveness to sodium nitrite supplementation. Of the 3,063 metabolites measured at baseline, at least one and, in many cases, several metabolites within a molecular class, predicted a subject's responsiveness to sodium nitrite intervention within each functional test (Table 7).

**Table 7:** Metabolic pathways with metabolites predicting responsiveness to 10 weeks of sodium nitrite supplementation

Molecular Class	Number of Predictor Molecules by Pathway
**Knee Flexor Rate of Torque Development**	
Glycerophospholipids	14
Sphingolipids	1
Carboxylic Acids	1
Indoles and Derivatives	1
Fatty Acyls	1
Unknown	26
**Knee Extensor Rate of Torque Development**	
Glycerophospholipids	2
Sphingolipids	1
Carbohydrates and Carbohydrate Conjugates	2
Unknown	26
**Rapid Step**	
Glycerophospholipids	2
Carbonyl Compounds	1
Carboxylic Acids and Derivatives	1
Unknown	11
**Trail Making Test B**	
Glycerophospholipids	2
Glycerolipids	1
Unknown	14

Improvements in knee-extensor RTD with sodium nitrite were significantly related to baseline concentrations of 31 molecules belonging to three known molecular classes (specific molecules or molecular weights are listed in Supplemental Table 3), whereas gains in knee-flexor RTD was significantly related to 44 metabolites populating five identified molecular classes (Supplemental Table 4). The molecular classes predicting an individual subject's RTD response to intervention included glycerol-phospholipid, sphingolipid, carboxylic acid, indole, fatty acyl and carbohydrate metabolism. Reduced occurrence of stepping errors in the rapid step test was related to small molecules within glycerophospholipid, carbonyl and carboxylic acid metabolite classes (Supplemental Table 5). Lastly, concentrations of 17 metabolites at baseline were significantly associated with individual improvements in cognitive function (TMT-B) in response to sodium nitrite (Supplemental Table 6).

## DISCUSSION

The pilot study yielded three key findings. First, 10 weeks of sodium nitrite supplementation in healthy, highly functioning MA/O adults increased plasma nitrite levels and improved several measurements of motor and cognitive function compared with placebo control. Second, sodium nitrite altered the plasma metabolome, with 75 metabolites exceeding our specified threshold for meaningful change in the two dosage groups. Importantly, the changes in 13 of these metabolites were independently related to the improvements in motor and cognitive outcomes, and the abundance of 99 metabolites detected at baseline predicted responsiveness to sodium nitrite supplementa-tion. Finally, improvements in function and changes in the plasma metabolome generally were observed in both the low- and high-dose groups, indicating that the lower dose of sodium nitrite is sufficient to obtain physiological benefits. Overall, these findings in healthy MA/O men and women provide critical preliminary evidence for conducting a larger clinical trial of sodium nitrite supplementation on motor and cognitive function and the plasma metabolome in older adults and groups with existing functional impairments.

### Efficacy: Motor and cognitive function

This pilot investigation demonstrates the efficacy of sodium nitrite supplementation for enhancing motor and cognitive functions in healthy MA/O adults with normal baseline function. These observations support a possible role for sodium nitrite supplementation in maintaining physiological function with aging in not only healthy MA/O adults, but also older and other groups with baseline functional impairments.

To our knowledge, the present results are the first to show that chronic supplementation with sodium nitrite can improve RTD in MA/O adults; a previous study reported improvements in young adults in response to acute supplementation of dietary nitrate [29]. Our novel observations in MA/O adults are potentially clinically important given that aging is associated with a progressive reduction in RTD [30, 31], which is related to adverse outcomes including loss of balance and falls [32]. Though no clinical cut points exist for RTD, age-related declines of 25-40% have been reported in healthy adults for knee flexion and extension RTD [31].

The present trial demonstrated 24-43% improvements in knee flexion and extension RTD in response to sodium nitrite supplementation, suggesting that much of the presumed decline in function with age was reversed [33, 34]. The age-related declines in RTD are largely dependent on reductions in the rate of muscle activation [35-37]. As such, sodium nitrite may have improved RTD via changes in neural mechanisms; this will be a key goal of future investigations.

The improvements observed in the TMT-B, an index of executive function, and the number of errors during the rapid step test, are consistent with a positive influence of sodium nitrite on neural/cognitive actions in MA/O adults. Performance on the TMT-B decreases with advancing age [38, 39] and in early stages of cognitive decline [40]. Although the average TMT-B time at baseline of 57.6 ± 17.3 s for our subjects already was ∼10% faster than the previously published age- and education-matched normative value of 64.6 ± 18.6 s [39], sodium nitrite supplementation nevertheless reduced the time to complete the task to those reported for healthy subjects 25-34 years of age [39]. The rapid step test also represents a challenging cognitive choice-reaction task [7, 41]. These findings are in agreement with a recent study demonstrating improvements in reaction time in type 2 diabetics following dietary nitrate supplementation [42], but contrasts with the results from a study of short-term dietary nitrate on computerized cognitive test performance [43]. Collectively, the present findings and those from these previous trials underscore the importance of duration of the supplementation period and test selection in future clinical trials. Given this clustering of positive neural/cognitive results with sodium nitrite supplementation, future clinical trials will need to expand the scope of cognitive functions assessed and use fMRI to understand potential neural mechanisms *in vivo*.

In addition to the potential neural/cognitive effects of sodium nitrite are the findings of improved cardiorespiratory exercise capacity in the 80 mg/day sodium nitrite group. The moderate improvement in treadmill time to exhaustion in this population is in agreement with our recent observation of marked improvements in endurance rota-rod running performance in old mice following 8 weeks of supplementation with sodium nitrite [21]. Improvements in endurance and exercise tolerance during large muscle activity have also been reported in studies in young adults following ingestion of sodium nitr*a*te or beetroot juice, even in the absence of changes in VO_2_max, heart rate, ventilation and other physiological responses [16-18, 44, 45], although no effects have been reported in some studies [43]. We also found a strong trend for improvement in heel-rise time in the low dose group, suggesting enhanced muscle endurance. The 33 s improvement in heel-rise in the present study with low-dose supplementation appears highly significant given observations of heel-rise performance declining by ∼30 s with aging in healthy adults [46]. In contrast, 400-m walk time did not improve, though this is likely due to the fast baseline walk times in all subjects indicative of a ceiling effect. Overall, these findings support the idea of conducting a larger trial using the lower (80 mg/day) dose.

Though a detailed description of the potential mechanisms by which motor and cognitive functions were improved is beyond the scope of this pilot investigation, some speculation may be helpful for designing future trials. One possibility is that the cognitive/neural functional improvements were mediated, at least in part, by neuromuscular effects, as age-related motor dysfunction has a neurological, not only muscular, etiology [47-49]. Although 10 weeks of supplementation is unlikely to affect age-related loss of motor neurons, enhanced neural signaling and activation of muscle are possible via NO's role as a multifunctional messenger molecule and regulator of cerebrovascular function [50-53]. There also are strong associations between vascular and cognitive function with age [54], and sodium nitrite supplementation reverses age-related vascular endothelial dysfunction in old animals [55]. Further, reduced NO bioavailability and signaling has been implicated in disrupted neurovascular coupling in which cerebrovascular blood flow is no longer matched to regional metabolic demand, thereby destabilizing neurons, synapses and neurotransmission [53, 56]. Consistent with this idea, evidence of improved coupling of blood flow to visual stimuli with co-administration of nitrate and the carbonic anhydrase inhibitor acetazolamide has been shown in young men [57]. Lastly, in preclinical studies in mice, we have demonstrated that chronic low-grade inflammation, as indicated by elevated tissue expression of pro-inflammatory cytokines, is a major mechanism contributing to vascular and motor dysfunction with aging [58, 59], and that sodium nitrite supplementation reverses age-related increases in vascular and skeletal muscle inflammation in these models [55, 59]. Moreover, increases in pro-inflammatory cytokines are associated with motor dysfunction and disability in older adults [5, 60], as well as with reduced cognitive performance with aging [61, 62]. Thus, it is possible that reductions in inflammation played a role in improved cognitive and/or motor performance with sodium nitrite supplementation in the present study.

### Clinical markers and metabolomics

In the present study, sodium nitrite administration markedly raised plasma nitrite concentrations acutely, but only modest increases in plasma nitrite were observed with chronic supplementation. This was expected given the short half-life of the compound [63]. Sodium nitrite treatment did not influence general subject characteristics including body weight, body composition, or clinical blood markers (fasting glucose, insulin or lipids). The latter suggests that improvements in function with sodium nitrite supplementation were not secondary to changes in these factors.

To gain initial insight into the molecular events related to improvements in motor and cognitive function with sodium nitrite treatment, we analyzed the composition and abundance levels of metabolites in the circulating plasma [64, 65]. This approach has been used previously, albeit in a limited manner, to assess the associations between changes in circulating metabolites and improvements in physical and/or metabolic function in response to interventions such as weight loss, bariatric surgery, and resistance exercise training [66-68].

The first goal of the present analysis was to determine if the intervention altered the plasma metabolome compared with placebo control. We found that sodium nitrite supplementation induced numerous changes in the plasma metabolome at each of the two doses. Changes in several of the metabolites were common to both doses, with additional metabolites modulated primarily by the low (80 mg/day) dose. The main molecular class changed by the two sodium nitrite doses was the glycerophospholipids. Previous research has shown that glycerophospholipid pathways are altered with advancing age [69], and that some of these pathways are associated with changes in the biological processes underlying aging, such as inflammation and oxidative stress. Our analysis demonstrated that sodium nitrite supplementation altered numerous subclasses of glycerophospholipids including lysophosphatidyl-cholines (LysoPCs), a group of pro-oxidant and inflammatory metabolites known to increase with advancing age and to be associated with declines in locomotor activity [70-73]. Both doses of sodium nitrite supplementation reduced the concentrations of LysoPCs and may partially explain the improvements in motor and cognitive outcomes. Other molecular classes influenced by both dose groups included sphingo-myelins, carboxylic acids and pyridines, which are implicated in inflammatory pathways, protein synthesis, and NAD+ metabolism, respectively [74-78].

A second goal of our analysis was to determine the relation, if any, between changes in individual metabolites or categories of metabolites with treatment and corresponding improvements in motor or cognitive function. The changes in the prioritized functional outcomes (knee flexor/knee extensor RTD, executive function and rapid step test errors) were significantly related to changes in selective metabolites, although the exact identity of approximately half of these molecules remain unknown and await future classification. For example, two unknown metabolites (C28 H44 O4 and C26 H58 N2 O2 S2) were significantly related to both knee flexor and extensor RTD in the 80 mg/day nitrite group, suggesting a possible link to the rate of muscle force production. Additionally, the relation of several glycerophospholipids and, more specifically, LysoPCs, with several functional outcomes indicate that some of the benefits associated with sodium nitrite supplementation may occur through changes in inflammation, as shown recently by our group in older mice [21]. Overall, these findings suggest that changes in small molecules related to lipid metabolism, inflammation and oxidative stress may reflect modifications in metabolic pathways that are involved in mediating improvements in physiological function with sodium nitrite supplementation. Further investigations into these associations may lead to the identification of novel pathways that can be targeted to improve physiological function in MA/O adults.

The final aim of the metabolomics analysis was to identify concentrations of metabolites at baseline that predict improvements in motor or cognitive function with treatment among subjects. Utilizing metabolomics to understand variability among individuals in the physiological responses to therapeutic interventions can provide novel insight into the differing mechanisms by which function is suppressed with aging, and to identify the most effective therapeutic strategies for individuals in the context of precision (personalized) medicine. Indeed, metabolomics has been used previously as such an approach in studies of cardiovascular disease [79], insulin sensitivity [67], depression [80] and cancer [79]. Applying similar techniques, we were able to identify metabolites from numerous molecular classes that showed an ability to explain the variation in responsiveness to sodium nitrite. Although combinations of specific molecular classes created unique predictive profiles for each functional measure, glycerophospholipids remained the most common class of molecules to predict responsiveness in the various functional domains. This observation is consistent with the results of our other analyses, indicating that glycerophospholipid metabolism may be a central target of sodium nitrite supplementation.

### Limitations

There are limitations in this pilot trial that should be acknowledged. The population was largely Caucasian, although the Hispanic and African American subjects who underwent sodium nitrite treatment appeared to respond similarly to the Caucasian subjects. As emphasized earlier, our subjects were healthy MA/O adults. However, it is possible that healthy older adults and/or MA/O patients with more impaired function at baseline (e.g., those with frailty/functional limitations, physical disability or mild cognitive impairment) could demonstrate even more robust responses to sodium nitrite supplementation.

Comprehensive interrogation of the mechanisms mediating the beneficial effects of sodium nitrite on function was beyond the scope of this first pilot study in human subjects, but an extensive analysis of the plasma metabolomics was performed to provide initial insight. Several of the metabolites demonstrating change with supplementation, including some that were associated with improvements in functional outcomes, were of unknown origin. Although new metabolites are continually being identified, it is not possible at present to identify all metabolites detected in an untargeted analysis [81, 82]. Moreover, our metabolomics analysis did not directly measure metabolic flux using labeled isotope techniques. Nevertheless, we believe that the use of a randomized, placebo controlled study design, and the quality control measures in our analysis allowed us to show changes in small metabolites in response to sodium nitrite supplementation.

### Conclusions

This pilot study provides the first evidence that chronic sodium nitrite supplementation improves selective measures of motor and cognitive function in healthy MA/O adults with good functional status at baseline. Importantly, we also show that sodium nitrite treatment changes numerous metabolic signatures of the plasma metabolome, that improvements in function are related to specific changes in small metabolites, and that a large number of metabolites at baseline predict functional responsiveness to this intervention. Overall, these results suggest that along with healthy lifestyle behaviors such as regular physical activity, sodium nitrite holds promise as a complementary pharmacological strategy for maintaining motor and cognitive function with aging, and provides an experimental basis for conducting a larger clinical trial of sodium nitrite supplementation in this and other MA/O populations.

## METHODS

### Subjects

A total of 86 MA/O (age 50-79 years) adults were recruited from the community using local newspaper and radio advertisements, university email advertisements and direct mailings. After comprehensive health screening at our Clinical Translational Research Center (CTRC) at the University of Colorado Boulder, 53 candidates were excluded from the study and the remaining 33 were enrolled. The enrolled subjects were all non-diabetic, had BMI <40 kg/m^2^, were cognitively intact as indicated by a Mini Mental Status Exam score >25 and were healthy and free of clinical diseases as assessed by medical history, physical examination, blood chemistry and resting and maximal exercise electrocardiogram (ECG) and blood pressure (BP) responses. Subjects were not taking cardiovascular acting drugs, drugs metabolized by the same liver enzymes as sodium nitrite, CNS depressants or drugs affecting neurological health. Women were postmenopausal for at least 1 year and had not taken hormone replacement therapy for at least the previous 6 months. The minority-ethnicity status of the subjects was 28 non-Hispanic white, 3 Hispanic white and 2 non-Hispanic black/African American.

All study procedures complied with the Declaration of Helsinki and the informed consent and study documents were approved by the Institutional Review Board of the University of Colorado Boulder and the Scientific Advisory and Research Committee of the University of Colorado Denver. The nature, benefits and risks of the study were explained to the volunteers and their written informed consent was obtained prior to participation.

### Study procedures

Measurements were performed at the University of Colorado Boulder CTRC and Neurophysiology of Human Movement Laboratory. The CTRC measures of subject characteristics and the plasma metabolome were obtained following 12-h overnight fast and 24-h abstention from alcohol and exercise. Measures of motor and cognitive function were obtained 2 h after ingesting a small snack, and following 24-h abstention from alcohol.

Following screening and baseline measures, subjects were randomized to low-dose (40 mg capsules, 2x/day) or high-dose (80 mg capsules, 2x/day) sodium nitrite or placebo (0 mg capsules, 2x/day) for 10 weeks. Adherence and safety were assessed at days 1 and 2, and weeks 4, 8 and 10 via safety questionnaire, monitoring of methemoglobin and blood pressure. Detailed information regarding adherence, safety and tolerability are found elsewhere [25]. Following 10 weeks of treatment, baseline study procedures were repeated. During the 10-week intervention/control period, 1 subject dropped out of the high-dose sodium nitrite group and 1 dropped out of the placebo control group. One subject in the low-dose group was excluded after positive smoking status was revealed. Therefore 30 subjects completed the study: 10 in each of the 3 groups.

### Subject characteristics, blood assays and dietary analysis

BMI (body mass index) was calculated from height and weight to the nearest 0.1 kg. Total body fat, total lean mass and regional lean mass were determined using dual energy X-ray absorptiometry (DEXA, GE/Lunar) [83]. Diet composition was estimated from 3-day food intake records (The Food Processor 8.2; ESHA Research) analyzed by a CTRC bionutritionist to assess and control for nitrate and nitrite in the diet. Fasting serum concentrations of glucose, insulin and total cholesterol were determined using standard assays performed by the University of Colorado Denver Anschutz Medical Campus CTRC core laboratory. Habitual physical activity was assessed at baseline from estimates of daily energy expenditure during leisure time and occupational activities, as previously described [84, 85], and estimated total kcals and total steps were measured objectively during 7-day (5 weekday, 2 weekend day) monitoring using Actigraph monitors (wGT3X-BT).

Acute and chronic levels of plasma nitrite were assessed by HPLC [86]. Acute levels were obtained 30 min following a single (initial) dose. Chronic levels were assessed at 10 weeks of sodium nitrite or placebo, 12-18 h after the most recent dose.

### Motor and cognitive function

Motor and cognitive function was assessed during a single, ∼3-h session. Motor function was assessed by measures of muscle strength and rate of force development, balance, fatigability, mobility and dexterity, and two simple tests of cognition, Trail Making Tests part A and B, also were obtained, along with questionnaires related to fatigue. Subjects were allowed ample rest time between the tests of motor function to ensure safety and adequate recovery from the previous task.

### Motor function outcomes

#### Rate of torque development and muscle strength

Rate of torque development (RTD) and maximal voluntary contractions (MVCs) were measured during fast or slow isometric contractions for the knee extensors and flexors of each subject as described in detail previously [87]. During the RTD task, subjects were instructed to perform 5-10 isometric contractions, with each contraction performed as quickly and forcefully as possible, but without holding the contraction at maximal torque; the peak torque per unit time (N·m/s) was recorded and calculated offline as the linear slope of the torque-time curve from 10-90% of the fast contractions. MVC was measured as peak torque (N·m) during 3-5 slow isometric contractions [87, 88]. Subjects also performed grip strength MVC with a standard dynamometer (hydraulic hand dynamometer, 300 lb capacity, Baseline Evaluation Instruments).

#### Cardiorespiratory fitness

Subjects performed incremental treadmill exercise using a modified Balke protocol as previously described [84] during a separate visit to the CU-Boulder CTRC. Oxygen consumption was measured using on-line, computer-assisted, open circuit spirometry during submaximal and maximal (VO_2_max) exercise workloads. Heart rate and ratings of perceived exertion (RPE) were measured throughout exercise and treadmill endurance was recorded as total exercise time to exhaustion as determined by subject's desire to stop despite strong verbal encouragement.

#### Fatigability and fatigue

Performance fatigability was assessed as the time to failure during a heel-rise test. The heel-rise test is a single-leg test in which subjects perform plantar flexion contractions at a rate of one maximal height heel-rise every 2 s until task failure [89]. The test was terminated when the subject voluntarily stopped due to an inability to continue or discomfort, used more than fingertips for balance during 2 consecutive heel raises, or decreased the required plantar flexion range of motion by more than 50%. Fatigue was assessed as a trait characteristic via two questionnaires: the Fatigue Severity Scale (FSS) and a brief Fatigue Questionnaire [90].

#### Balance

Balance was measured as the time and number of errors committed during a rapid step test [7]. Prior to the rapid step test, the maximal step length was determined and standardized across pre- and post-intervention conditions. Subjects completed three trials of rapid stepping to a random leg-direction (right or left leg: front, side, back) to taped targets placed on the floor corresponding to 80% of the maximal step length for each leg and direction as described previously [7]. An error was defined as failure to step beyond the target, loss of balance as indicated by the need for assistance to prevent a fall, failure to return to initial position, multiple steps, or noncompliance with a leg or direction command.

#### Mobility

Mobility was quantified with measures of endurance walk and Timed Up and Go. Walking endurance was assessed as the time to walk 400 m [27]. Subjects walked at a brisk pace for 2.5 laps around an indoor track as described previously [87, 91]. Mobility was also quantified by a Timed Up and Go task as the time to stand unassisted, walk to a target located 3 m from the chair, and return to a seated position [92].

#### Manual dexterity

Manual dexterity was measured as the time to complete the Grooved Pegboard test, a standardized test of manual dexterity that is known to decline in older compared with middle-aged and young adults [2]. Subjects were instructed to place the 25 keyhole-shaped pegs into the holes of a pegboard (Lafayette Instruments) as quickly as possible. All subjects completed three trials and the average and fastest times were recorded [2].

### Cognitive outcomes

Trail Making Tests A and B (TMT-A and TMT-B) were administered according to guidelines [39, 93]. Participants were instructed to complete each part of the TMT as quickly and accurately as possible. When an error was made, the participant was instructed to return to the “circle” where the error originated and continue. Time to complete each part was recorded.

### Metabolomics

EDTA-treated plasma was collected before and after 10 weeks of sodium nitrite or placebo treatment, frozen and stored at −80°C until analysis. Compounds were extracted from samples using an in-house developed technique that yields molecules from 4 major classes (lipid, protein, carbohydrate and nucleic acids) [94-96]. Aqueous and lipid fractions were analyzed using Liquid Chromatography Mass Spectrometry (LCMS, Agilent 6210 ESI-TOF) and data analyzed using commercial and in-house developed software [96-98]. Quality control included duplicate samples and analysis of a pooled plasma sample containing labeled or exogenous negative or positive spike-in controls, resulting in false discovery rates <3%. Spectral data was extracted and aligned based on mass and retention time using Profinder software (Agilent). Metabolites were annotated using IDBrowser in Mass Profiler Professional (MPP, Agilent) using a combination of available databases, including METLIN, Human Metabolome Database (HMDB), LipidMaps, and Kyoto Encyclopedia of Genes and Genomes (KEGG). Metabolites identified as being significantly altered by treatment were confirmed using MS/MS (Agilent 6520 Q-TOF) and matched to the NIST 14 MSMS spectral library, as shown previously [99]. Chemical formula generation was attempted for all molecules that remained unidentified in both LC-MS and LC-MS/MS analyses.

### Statistics

#### General statistics

Data are presented as mean ± SEM (figures) or mean ± SD (tables and text). Statistical analysis was performed with SPSS software (v21, IBM). Prior to primary analysis of the motor function outcomes, normality of each variable was assessed with the Kolmogorov-Smirnov test [87]. No outliers (>3 SD) were identified for either physical or cognitive outcomes. Homogeneity of variance was assessed by Levene's test and did not differ among groups (p>0.05). Subject characteristics and outcomes of interest were compared at baseline via one-way ANOVA. Group differences in motor and cognitive functions with sodium nitrite treatments compared with placebo was determined by mixed-model ANOVA (within factor, time; between factor, treatment group), with contrast set to changes observed in placebo controls, and followed by post-hoc Tukey's HSD means-comparison. The α-level for significance was 0.05; however, as this was a pilot investigation being used to determine outcomes of interest for a larger parent trial, trends are also reported with an α-level of p < 0.1.

#### Metabolomics analysis

Metabolomics data were filtered for analysis using Mass Profiler Professional (Agilent). Small molecules not present in at least 50% of any one group before or after the intervention were excluded to reduce the probability of false discovery, resulting in 3,063 detected small molecules for subsequent analyses. Molecular concentrations were transformed to a log_2_ scale to assess their total magnitude of change. To detect metabolites significantly altered by treatment, small molecules were required to withstand a two-fold change filter from baseline and remain significant after a paired t-test (p<0.05), while not being significantly (p<0.05) altered in the placebo group. Based upon a database search, metabolites known to be attributed to dietary consumption of foods were excluded from analysis. The analysis resulted in 45 and 33 metabolites being significantly altered in the 80 mg and 160 mg groups, respectively. These molecules were then modeled independently using linear regression within each treatment group to assess their association with functional outcomes. Only metabolites significantly associated with functional measures are reported.

To assess whether baseline metabolomic signatures can predict responsiveness to sodium nitrite intervention, log_2_ concentrations of all 3,063 molecules detected at baseline were tested against responsiveness to nitrite intervention using independent linear regression models. Only metabolites that were significantly associated with responsiveness to the intervention and did not show an interaction with dosage are reported. Metabolites were grouped by class using the HMDB database to assess which pathways were most affected by sodium nitrite. A subject was determined as being responsive to intervention in each functional measure if they exhibited a ≥10% improvement from baseline.

## SUPPLEMENTARY DATA FIGURES AND TABLES


