# Supplementary material for: Improved motor and cognitive performance with sodium nitrite supplementation is related to small metabolite signatures: a pilot trial in middle-aged and older adults
Source: Aging (Albany NY). 2015 Nov 30;7(11):1004–21. doi: 10.18632/aging.100842 (PMC4694069; doi:10.18632/aging.100842)
Supplement: Supplementary file 1 [file aging-07-1004-s001.docx]

**SUPPLEMENTAL DATA**

**Supplemental Table 1**. Metabolites significantly altered by 80 mg/day of sodium nitrite supplementation (2 fold-change, p<0.05).

| **Glycerophospholipids** |  | | | |
| --- | --- | --- | --- | --- |
| Compound | Mass | Retention Time | P-Value | FC |
| PS(25:0) | 637.4633 | 4.321999 | <0.0001 | -2.1170917 |
| LysoPE(18:3(9Z,12Z,15Z)/0:0) | 475.2687 | 1.6749995 | <0.0001 | 2.1252353 |
| PC(38:2) | 813.6039 | 2.9290006 | <0.0001 | -3.2069693 |
| PG(20:2/13:0) | 754.4724 | 1.1299996 | <0.0001 | -9856.007 |
| PE(42:4) | 823.5323 | 5.265999 | 0.001837377 | -557.9828 |
| LysoPC(18:1) | 999.1222 | 2.0549989 | 0.012707732 | -4.9534955 |
| LysoPC(18:0) | 1046.2844 | 2.4279988 | 0.024706118 | -3.835653 |
| PC(16:0/22:6) | 1607.5673 | 5.571006 | 0.025083639 | -2.3075466 |
| PC(20:4) | 769.5907 | 6.446007 | 0.025124883 | -749.48114 |
| PI(38:5) | 884.5405 | 5.553002 | 0.029784223 | -55.981827 |
| PI(38:6) | 882.5269 | 5.0650015 | 0.033713326 | -59.240566 |
| PC(18:0/18:1) | 809.8326 | 6.431 | 0.042184845 | 134.62251 |
| PC(16:0/18:1) | 744.6018 | 6.3489995 | 0.04979071 | 86.89184 |


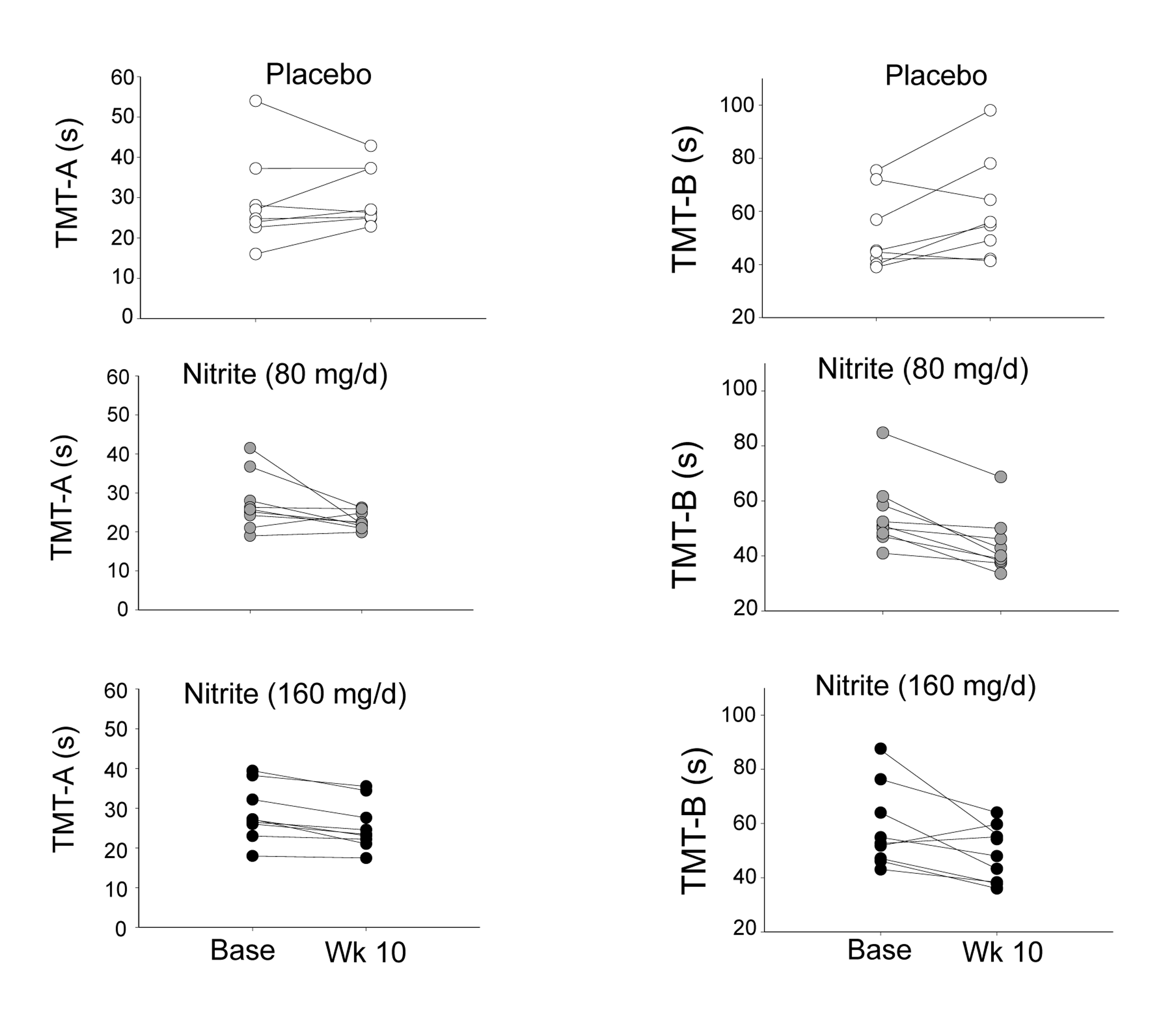


**Supplemental Figure 1.** Individual subject data indicated that time to complete the Trail Making Test-A (Left panel) and -B (Right panel) was variable across 10 weeks for on placebo (white circles), but improved, as demonstrated by a subtle decline in time in majority of subjects taking sodium nitrite 80 mg/day (gray circles), and subjects supplemented with 160 mg/day (Trail Making Test-B only; black circles).

| **Sphingolipids** | | | | | | | | | | | |
| --- | --- | --- | --- | --- | --- | --- | --- | --- | --- | --- | --- |
| Compound | | Mass | | Retention Time | | | P-Value | | | FC | |
| SM(d18:1/24:1) | | 812.677 | | 7.474 | | | 0.016271695 | | | -1319.5327 | |
| SM(d18:1/16:0) | | 702.7867 | | 4.782998 | | | 0.017466838 | | | -2.4910555 | |
| **Glycerolipids** | | | | | | | | | | | |
| Compound | | Mass | | Retention Time | | P-Value | | | FC | |  |
| DG(28:2) | | 525.261 | | 0.60199976 | | <0.0001 | | | 22.427322 | |  |
| **Fatty Acyls** | |  | | | | | | | | | |
| Compound | | Mass | | Retention Time | | | P-Value | | | FC | |
| 9-Decenoylcarnitine | | 313.2258 | | 3.7760007 | | | <0.0001 | | | -16 | |
| Docosanamine | | 325.3717 | | 0.7320002 | | | <0.0001 | | | -2.23001 | |
| β-Methylcrotonyl coenzyme A | | 849.6558 | | 7.756002 | | | 0.033463486 | | | -107.49303 | |
| **Carbohydrates and Carbohydrate Conjugates** | | | | | | | | | | | |
| Compound | | Mass | | Retention Time | | | P-Value | | | FC | |
| Chondroitin 4-sulfate | | 512.3537 | | 1.3100004 | | | <0.0001 | | | -11.16415 | |
| **Carboxylic Acids and Derivatives** | | | | | | | | | | | |
| Compound | | Mass | | Retention Time | | | P-Value | | | FC | |
| N,N-Dimethyl-L-valine | | 145.0707 | | 5.4290004 | | | <0.0001 | | | -8223.001 | |
| Dopaquinone | | 195.0559 | | 6.175998 | | | <0.0001 | | | 16.637007 | |
| **Imidazole Ribonucleosides and Ribonucleotides** | | | | | | | | | | | |
| Compound | | Mass | | Retention Time | | | P-Value | | | FC | |
| 5-Aminoimidazole-4-carboxamide-1-βD-ribofuranosyl 5'-monophosphate | | 676.7675 | | 4.632998 | | | 0.006778782 | | | -2.2249255 | |
| **Pyridines and Derivatives** | | | | | | | | | | | |
| Compound | | Mass | | Retention Time | | | P-Value | | | FC | |
| 1-Methyl-2-pyridone-5-carboxamide | | 337.3316 | | 0.514 | | | 0.025208822 | | | 2.207129 | |
| **Peptide Hormones** | | | | | | | | | | | |
| Compound | | Mass | | Retention Time | | | P-Value | | | FC | |
| Secretin | | 2003.8461 | | 1.9730009 | | | 0.048196305 | | | -2.0949135 | |
| Secretin | 2003.8461 | | 1.9730009 | | 0.048196305 | | | -2.0949135 | | | |

| **Unknown** |  | | | |
| --- | --- | --- | --- | --- |
| Compound | Mass | Retention Time | P-Value | FC |
| C26 H58 N2 O2 S2 | 494.3947 | 4.019999 | <0.0001 | -3.6539412 |
| C37 H59 N P4 | 641.3691 | 2.0120006 | <0.0001 | -31.826004 |
| C22 H23 F N6 | 390.1983 | 0.625 | <0.0001 | -13.65919 |
| C17 H18 N2 O4 | 314.1259 | 0.96400017 | 3.08E-04 | -2.0077014 |
| C9 H16 N2 O6 | 248.1051 | 6.464998 | 0.001024577 | -106.081245 |
| C18 H18 N4 O6 S4 | 496.0008 | 1.9769999 | 0.003148865 | 5.026388 |
| C35 H62 N O15 P | 1572.6686 | 6.567995 | 0.004305141 | -2.2063572 |
| C16 H6 N O18 P3 S4 | 720.7684 | 4.6229978 | 0.005506006 | -2.1636457 |
| C35 H14 N9 O11 P | 767.0537 | 6.0620036 | 0.008039862 | -2.670254 |
| C47 H96 | 1441.5406 | 4.6260037 | 0.009113913 | -2.271578 |
| C28 H44 O4 | 444.327 | 2.7180014 | 0.014350595 | -146.8961 |
| C37 H58 N3 P3 | 637.384 | 4.041998 | 0.018353136 | 1088.5378 |
| C51 H6 N P3 S | 753.7664 | 5.1340017 | 0.019845642 | -130.73598 |
| C22 H22 O14 S | 524.0608 | 2.440999 | 0.022118837 | -2.1691334 |
| C14 H29 N16 O12 P3 S2 | 769.5712 | 6.213996 | 0.035132013 | -86.339264 |
| C27 H32 N12 O P2 S5 | 760.0992 | 5.9549947 | 0.038911335 | -96.27765 |
| C4 H6 Cl2 O15 P2 S3 | 521.764 | 2.0599988 | 0.042757154 | -2.1440578 |
| C66 H107 N16 P | 1154.8597 | 4.980999 | 0.04323044 | -68.32851 |
| C76 H131 Cl2 N17 P2 S4 | 1541.6263 | 5.7839975 | 0.04371927 | -2.2895992 |
| C19 H50 N11 O13 P S | 703.301 | 5.3429956 | 0.04401792 | -2.040719 |

**Supplemental Table 2**. Compounds significantly altered by 160 mg/day of sodium nitrite supplementation (2 fold-change, p<0.05).

| **Glycerophospholipids** |  | | | | |
| --- | --- | --- | --- | --- | --- |
| Compound | Mass | Retention Time | | P-Value | FC |
| PC(P-19:1/0:0) | 1001.0781 | 1.9839997 | | 0.021031061 | -61.77204 |
| PE(28:6) | 623.3154 | 3.8020015 | | 0.035558406 | -57.265976 |
| PE(P-16:0/20:4) | 723.5173 | 5.829003 | | 0.038817443 | 48.740414 |
| PC(38:4) | 1661.1422 | 6.2529964 | | 0.03925534 | -217.92023 |
| LysoPC(20:5) | 541.516 | 1.6340011 | | 0.040064152 | -32.21305 |
| PE(22:5) | 541.5351 | 1.9660017 | | 0.04191952 | -2.317909 |
| PC(18:1/18:1) | 807.8034 | 5.578002 | | 0.042040516 | 2.044843 |
| PE(30:0) | 663.4803 | 5.5910025 | | 0.04857921 | 2.101735 |
| PC(18:4(6Z,9Z,12Z,15Z)/14:1(9Z) | 242.1692 | 0.5889998 | | <0.0001 | -16 |
| **Sphingolipids** |  | | | | |
| Compound | Mass | | Retention Time | P-Value | FC |
| SM(d18:1/24:1) | 812.677 | | 7.474 | 0.03466193 | 497.0157 |
| SM(d18:1/24:0) | 836.0701 | | 6.458006 | 0.003240414 | -397.12375 |

| **Carboxylic Acids and Derivatives** | | | | |
| --- | --- | --- | --- | --- |
| Compound | Mass | Retention Time | P-Value | FC |
| L-Glutamine | 129.0467 | 5.964998 | <0.0001 | 16 |
| Creatine | 131.0696 | 4.7739973 | 0.032669824 | 295.365 |
| **Polypeptide** | | | | |
| Compound | Mass | Retention Time | P-Value | FC |
| Gonadotropin Releasing Peptide | 774.5662 | 5.0060024 | 0.016316624 | -183.44456 |
| **Pyridines and Derivatives** | | | | |
| Compound | Mass | Retention Time | P-Value | FC |
| Pyridoxamine | 151.0254 | 4.790001 | 0.029969934 | 91.74875 |
| **Unknown** | | | | |
| Compound | Mass | Retention Time | P-Value | FC |
| C28 H4 Cl N2 O32 P3 S5 | 1167.6179 | 6.326002 | <0.0001 | 9830.448 |
| C25 H10 Cl3 N3 O3 P2 | 566.9226 | 3.4240017 | <0.0001 | 11.123737 |
| C15 H43 N15 O2 S4 | 593.2649 | 3.8010006 | <0.0001 | -30.760254 |
| C42 H84N O9 P | 777.5849 | 2.726 | <0.0001 | 9.677231 |
| 421.4328@1.2670001 | 421.4328 | 1.2670001 | <0.0001 | 16 |
| C37 H59 N P4 | 641.3691 | 2.0120006 | <0.0001 | -5.943493 |
| C22 H23 F N6 | 390.1983 | 0.625 | <0.0001 | -10.188005 |
| C13 H27 N4 P | 270.1972 | 0.276 | <0.0001 | 79.5715 |
| C37 H58 N3 P3 | 637.384 | 4.041998 | 4.09E-07 | 9227.953 |
| C34 H53 N6 O2 P | 608.3976 | 4.257002 | 0.001038205 | 2.2397087 |
| C24 H50 N12 | 506.4269 | 2.939 | 0.005681379 | 2.3796062 |
| C35 H60 N2 O4 | 608.3834 | 5.2090044 | 0.025873546 | 37.549183 |
| C26 H58 N2 O2 S2 | 494.3947 | 3.4459994 | 0.030893583 | 108.87422 |
| C48 H93 Cl N6 O2 S | 852.6477 | 7.0199966 | 0.033704307 | -3.0958116 |
| C33 H68 O2 | 496.5263 | 1.9670002 | 0.03734944 | 3.1074245 |
| C11 H30 Cl2 N4 O12 P4 | 603.9426 | 1.2850013 | 0.03876037 | -20.8693 |
| C71 H120 N5 O P S2 | 1153.866 | 5.6099944 | 0.04072563 | 2.040464 |
| C8 H8 Cl3 N3 O4 S2 | 1607.6018 | 5.875 | 0.045195773 | -2.3322148 |

**Supplemental Table 3**. Baseline concentrations of specific metabolites predicting an individual’s functional res-

ponsiveness (≥10% improvement) in knee-extensor rate of torque development with sodium nitrite supplementation.

| **Glycerophospholipids** | |  |  |  |  | |  |
| --- | --- | --- | --- | --- | --- | --- | --- |
| Compound | Mass | Retention Time | Beta | Std. Error | | P-Value | |
| PS(22:4(7Z,10Z,13Z,16Z)/20:4(5Z,8Z,11Z,14Z)) | 859.5339 | 5.4349985 | 0.548 | 0.011 | | 0.012 | |
| PA(14:0/13:0) | 578.3891 | 3.8639987 | 0.706 | 0.011 | | 0.001 | |
| **Sphingolipids** | |  |  |  | |  | |
| Compound Name | Mass | Retention Time | Beta | Std. Error | | P-Value | |
| LacCer(d18:0/24:1) | 1017.7243 | 6.997003 | 0.547 | 0.021 | | 0.013 | |
| **Carbohydrates and carbohydrate conjugates** | | | |  |  | |  |
| Compound Name | Mass | Retention Time | Beta | Std. Error | | P-Value | |
| D-Glucose-13C6 | 208.0659 | 0.993 | 0.567 | 0.013 | | 0.009 | |
| Beta-D-Glucopyranuronic acid | 314.0642 | 6.7439127 | 0.554 | 0.016 | | 0.011 | |
| **Unknown** |  |  |  |  |  | |  |
| Compound Name | Mass | Retention Time | Beta | Std. Error | | P-Value | |
| C25 H55 N4 P S | 474.3628 | 3.4873846 | 0.731 | 0.012 | | <0.001 | |
| 1581.1028 @ 5.4700007 | 1581.1028 | 5.4700007 | 0.589 | 0.018 | | 0.006 | |
| C37 H77 N7 O2 | 651.613 | 7.9320045 | 0.542 | 0.021 | | 0.014 | |
| C42 H91 Cl2 N7 S2 | 827.63 | 6.9079957 | 0.568 | 0.019 | | 0.009 | |
| 3106.2756@6.0880046 | 3106.2756 | 6.0880046 | 0.56 | 0.015 | | 0.01 | |
| 1505.0986@5.950004 | 1505.0986 | 5.950004 | 0.549 | 0.148 | | 0.012 | |
| 1607.7668@5.604003 | 1607.7668 | 5.604003 | 0.526 | 0.096 | | 0.017 | |
| 3157.328@5.772002 | 3157.328 | 5.772002 | 0.561 | 0.102 | | 0.01 | |
| 761.1707@6.3710017 | 761.1707 | 6.3710017 | 0.592 | 0.071 | | 0.006 | |
| 774.5541@4.851998 | 774.5541 | 4.851998 | 0.704 | 0.01 | | 0.001 | |
| 1569.6495@6.4299946 | 1569.6495 | 6.4299946 | 0.532 | 0.01 | | 0.016 | |
| 1567.163@6.3779964 | 1567.163 | 6.3779964 | 0.687 | 0.01 | | 0.001 | |
| 625.8175 @ 4.1889977 | 625.8175 | 4.1889977 | 0.517 | 0.658 | | 0.02 | |
| 1904.7849@4.571004 | 1904.7849 | 4.571004 | 0.53 | 0.535 | | 0.016 | |
| 527.8407 @ 4.1889977 | 527.8407 | 4.1889977 | 0.541 | 0.784 | | 0.014 | |
| 2142.8767@4.571004 :4 | 2142.8767 | 4.571004 | 0.521 | 0.313 | | 0.019 | |
| 723.7948 @ 4.1889977 | 723.7948 | 4.1889977 | 0.521 | 0.688 | | 0.018 | |
| 821.7702 @ 4.1889977 | 821.7702 | 4.1889977 | 0.503 | 0.582 | | 0.024 | |
| C10 H26 N9 P | 303.2047 | 4.907003 | 0.542 | 0.02 | | 0.013 | |
| C17 H7 N3 O14 | 476.8797 | 4.5719986 | 0.481 | 0.24 | | 0.032 | |
| C44 H89 N7 O4 S2 | 843.645 | 3.3289986 | 0.549 | 0.011 | | 0.012 | |
| 714.7772 @ 4.5899973 | 714.7772 | 4.5899973 | 0.508 | 0.5 | | 0.022 | |
| C32 H66 N6 S2 | 598.4871 | 0.79800016 | -0.616 | 0.013 | | 0.004 | |
| 2380.9763@4.571004 :24 | 2380.9763 | 4.571004 | 0.546 | 0.31 | | 0.013 | |
| 398.2433 @ 0.33099988 | 398.2433 | 0.33099988 | 0.552 | 0.021 | | 0.012 | |
| 919.7438 @ 4.192996 | 919.7438 | 4.192996 | 0.517 | 0.469 | | 0.02 | |

**Supplemental Table 4**. Baseline concentrations of specific metabolites predicting an individual’s functional res-

ponsiveness (≥10% improvement) in knee-flexor rate of torque development with sodium nitrite supplementation.

| **Glycerophospholipids** | | | | | | | | | |
| --- | --- | --- | --- | --- | --- | --- | --- | --- | --- |
| Compound | Mass | | Retention Time | | Beta | | Std. Error | | P-Value |
| PS(21:0/22:6(4Z,7Z,10Z,13Z,16Z,19Z)) | 877.5801 | | 5.691995 | | 0.52 | | 0.224 | | 0.019 |
| PS(22:4(7Z,10Z,13Z,16Z)/21:0) | 881.6107 | | 6.4960017 | | 0.444 | | 0.262 | | 0.05 |
| PS(19:0/18:4(6Z,9Z,12Z,15Z)) | 797.5243 | | 4.6350007 | | 0.735 | | 0.013 | | <0.001 |
| PS(22:4(7Z,10Z,13Z,16Z)/20:4(5Z,8Z,11Z,14Z)) | 859.5339 | | 5.4349985 | | 0.551 | | 0.011 | | 0.012 |
| PG(22:1(11Z)/19:0) | 882.6143 | | 6.4960017 | | 0.446 | | 0.263 | | 0.048 |
| PA(14:0/13:0) | 578.3891 | | 3.8639987 | | 0.458 | | 0.014 | | 0.042 |
| PC(20:3(8Z,11Z,14Z)/20:4(8Z,11Z,14Z,17Z)) | 831.5769 | | 5.6820045 | | 0.489 | | 0.218 | | 0.029 |
| LacCer(d18:0/14:0) | 835.5922 | | 6.2929997 | | 0.568 | | 0.25 | | 0.009 |
| PC(20:3(5Z,8Z,11Z)/20:3(8Z,11Z,14Z)) | 833.5903 | | 2.778 | | 0.538 | | 0.252 | | 0.014 |
| PC(20:3(8Z,11Z,14Z)/20:4(8Z,11Z,14Z,17Z)) | 831.576 | | 2.5209973 | | 0.571 | | 0.361 | | 0.009 |
| PC(18:3(6Z,9Z,12Z)/20:0) | 833.5929 | | 2.778 | | 0.541 | | 0.254 | | 0.014 |
| PE(22:5(4Z,7Z,10Z,13Z,16Z)/22:5(7Z,10Z,13Z,16Z,19Z)) | 837.593 | | 2.688002 | | 0.573 | | 0.017 | | 0.008 |
| PC(20:4(5Z,8Z,11Z,14Z)/20:4(8Z,11Z,14Z,17Z)) | 829.5694 | | 2.522002 | | 0.66 | | 0.306 | | 0.002 |
| PC(22:5(7Z,10Z,13Z,16Z,19Z)/P-16:0) | 791.5757 | | 2.6269994 | | 0.582 | | 0.017 | | 0.007 |
| **Sphingolipids** | | | | | | | | | |
| Compound | Mass | | Retention Time | | Beta | | Std. Error | | P-Value |
| 3-O-Sulfogalactosylceramide (d18:1/22:0) | 863.6129 | | 2.5570018 | | 0.453 | | 0.517 | | 0.045 |
| **Carboxylic Acids** | | | | | | | | | |
| Compound | Mass | | Retention Time | | Beta | | Std. Error | | P-Value |
| Thiocysteine | 198.9996 | | 0.7970005 | | 0.617 | | 0.012 | | 0.004 |
| **Indoles and derivatives** | | | | | | | | | |
| Compound | Mass | Retention Time | | Beta | | Std. Error | | P-Value | |
| Tryptophanamide | 203.1051 | 0.9280616 | | 0.522 | | 0.012 | | 0.018 | |
| **Fatty Acyls** | | | | | | | | | |
| Compound | Mass | Retention Time | | Beta | | Std. Error | | P-Value | |
| 20-trifluoro-LTB4 | 390.1981 | 2.7140007 | | -0.649 | | 0.266 | | 0.002 | |
| **Unknown** | | | | | | | | | |
| Compound | Mass | Retention Time | | Beta | | Std. Error | | P-Value | |
| 567.8508@2.0650003 | 567.8508 | 2.0650003 | | -0.602 | | 0.012 | | 0.005 | |
| 821.5736@5.4920025 | 821.5736 | 5.4920025 | | -0.536 | | 0.237 | | 0.015 | |
| 945.5686@5.691995 | 945.5686 | 5.691995 | | 0.483 | | 0.255 | | 0.031 | |
| 809.5557@6.2760005 | 809.5557 | 6.2760005 | | 0.533 | | 0.179 | | 0.015 | |
| 805.0461@5.429995 | 805.0461 | 5.429995 | | 0.523 | | 0.012 | | 0.018 | |
| 1531.1726@6.6969967 | 1531.1726 | 6.6969967 | | 0.553 | | 0.016 | | 0.011 | |
| 1543.1083 @ 5.4819965 | 1543.1083 | 5.4819965 | | 0.475 | | 0.227 | | 0.034 | |
| 779.5449@5.540994 | 779.5449 | 5.540994 | | 0.526 | | 0.087 | | 0.017 | |
| 728.57@5.5339975 | 728.57 | 5.5339975 | | -0.473 | | 0.129 | | 0.035 | |

| 743.5486@5.5450044 | 743.5486 | 5.5450044 | 0.545 | 0.175 | 0.013 |
| --- | --- | --- | --- | --- | --- |
| 3157.328@5.772002 | 3157.328 | 5.772002 | 0.559 | 0.102 | 0.01 |
| C49 H97 N5 | 755.7795 | 5.3110027 | 0.513 | 0.111 | 0.021 |
| 745.0467@6.2349935 | 745.0467 | 6.2349935 | -0.517 | 0.11 | 0.019 |
| 326.2188@3.440998 | 326.2188 | 3.440998 | -0.45 | 0.326 | 0.046 |
| 630.4534@3.4450037 | 630.4534 | 3.4450037 | -0.516 | 0.215 | 0.02 |
| 782.914 @ 5.4819965 | 782.914 | 5.4819965 | 0.613 | 0.068 | 0.004 |
| C31 H68 N26 O2 | 836.6075 | 6.278007 | 0.491 | 0.323 | 0.028 |
| 676.6538@3.4349983 | 676.6538 | 3.4349983 | -0.527 | 0.323 | 0.017 |
| 436.2829@3.477998 | 436.2829 | 3.477998 | -0.507 | 0.438 | 0.023 |
| 797.5479@5.536996 | 797.5479 | 5.536996 | 0.525 | 0.187 | 0.017 |
| 1564.149@6.3039937 | 1564.149 | 6.3039937 | 0.458 | 0.01 | 0.042 |
| 1018.6866@2.431 | 1018.6866 | 2.431 | 0.521 | 0.012 | 0.019 |
| 774.5541@4.851998 | 774.5541 | 4.851998 | 0.487 | 0.013 | 0.029 |
| C45 H87 Cl2 N5 O3 | 815.6142 | 3.4129977 | 0.461 | 0.012 | 0.041 |
| 476.671@4.572 | 476.671 | 4.572 | 0.571 | 0.016 | 0.009 |
| C15 H8 O | 204.0581 | 1.0159994 | 0.569 | 0.02 | 0.009 |

**Supplemental Table 5**. Baseline concentrations of specific metabolites predicting an individual’s functional responsiveness (≥10% improvement) in rapid step test errors with sodium nitrite supplementation.

| **Glycerophospholipids** | |  | | | |  | |  |
| --- | --- | --- | --- | --- | --- | --- | --- | --- |
| Compound | Mass | Retention Time | Beta | | Std. Error | | P-Value | |
| PS(O-16:0/19:0) | 745.5566 | 6.0459967 | 0.655 | | 1.892 | | 0.002 | |
| LysoPC(20:5) | 541.516 | 1.6340011 | 0.575 | | 0.019 | | 0.01 | |
| **Carbonyl Compounds** | |  |  |  | |  | |  |
| Compound | Mass | Retention Time | Beta | | Std. Error | | P-Value | |
| N4-Acetylaminobutanal | 129.0786 | 11.129011 | -0.59 | | 0.29 | | 0.008 | |
| **Carboxylic Acids and Derivatives** | | |  |  | |  | |  |
| Compound | Mass | Retention Time | Beta | | Std. Error | | P-Value | |
| Asymmetric dimethylarginine | 202.1415 | 11.377995 | -0.569 | | 0.317 | | 0.011 | |
| **Unknown** |  |  |  |  | |  | |  |
| Compound | Mass | Retention Time | Beta | | Std. Error | | P-Value | |
| 494.3432@5.556 | 494.3432 | 5.556 | 0.756 | | 0.012 | | <0.001 | |
| 1465.5632 @ 5.718996 | 1465.5632 | 5.718996 | 0.561 | | 0.098 | | 0.013 | |
| C50 H14 N6 P2 | 760.0765 | 6.355005 | 0.563 | | 0.385 | | 0.012 | |
| 1583.148@6.2650065 | 1583.148 | 6.2650065 | 0.57 | | 0.167 | | 0.011 | |
| 550.4186@3.865998 | 550.4186 | 3.865998 | 0.62 | | 0.176 | | 0.005 | |
| C5 H8 O2 S | 132.0259 | 2.6829991 | -0.505 | | 0.223 | | 0.027 | |
| 1613.1467 @ 3.7869997 | 1613.1467 | 3.7869997 | 0.496 | | 0.238 | | 0.031 | |
| 1521.6335 @ 3.2539985 | 1521.6335 | 3.2539985 | 0.705 | | 0.23 | | 0.001 | |
| 1521.67 @ 3.2559984 | 1521.67 | 3.2559984 | 0.764 | | 0.236 | | <0.001 | |
| C32 H66 N6 S2 | 598.4871 | 0.79800016 | -0.556 | | 0.015 | | 0.013 | |
| 1521.6492 @ 3.2519999 :21 | 1521.6492 | 3.2519999 | 0.688 | | 0.275 | | 0.001 | |

**Supplemental Table 6**. Baseline concentrations of specific metabolites predicting an individual’s functional responsiveness (≥10% improvement) in Trail Making Test-B with sodium nitrite supplementation.

| **Glycerophospholipids** |  |  |  |  |  | |
| --- | --- | --- | --- | --- | --- | --- |
| Compound | Mass | Retention Time | Beta | Std. Error | | P-Value |
| LysoPE(0:0/20:3(8Z,11Z,14Z)) | 503.3002 | 1.9869998 | -0.541 | 0.15 | | 0.014 |
| LysoPE(0:0/22:4(7Z,10Z,13Z,16Z)) | 529.3169 | 2.1309984 | -0.558 | 0.157 | | 0.011 |
| **Glycerolipids** |  |  |  |  |  | |
| Compound | Mass | Retention Time | Beta | Std. Error | | P-Value |
| DG(28:2) | 525.261 | 0.60199976 | -0.457 | 0.015 | | 0.043 |
| **Unknown** |  |  |  |  |  | |
| Compound | Mass | Retention Time | Beta | Std. Error | | P-Value |
| C25 H55 N4 P S | 474.3628 | 3.4873846 | 0.551 | 0.016 | | 0.012 |
| 576.4701@3.2960024 | 576.4701 | 3.2960024 | -0.541 | 0.052 | | 0.014 |
| 1669.8085 @ 7.324001 | 1669.8085 | 7.324001 | 0.499 | 0.162 | | 0.025 |
| C108 H129 Cl N49 O19 P | 2481.975 | 7.3150015 | 0.522 | 0.13 | | 0.018 |
| 570.486@3.3130026 | 570.486 | 3.3130026 | -0.558 | 0.093 | | 0.011 |
| 813.0178 @ 7.325995 | 813.0178 | 7.325995 | 0.581 | 0.14 | | 0.007 |
| 592.47@3.2910001 | 592.47 | 3.2910001 | -0.542 | 0.089 | | 0.014 |
| 1528.1018@5.471001 | 1528.1018 | 5.471001 | 0.575 | 0.013 | | 0.008 |
| 360.9133 @ 4.218965 | 360.9133 | 4.2189965 | -0.444 | 0.322 | | 0.05 |
| C38 H68 Cl N11 O | 727.5205 | 2.819 | -0.445 | 0.013 | | 0.049 |
| C11 H12 N6 O10 | 388.0601 | 0.6300002 | -0.52 | 0.277 | | 0.019 |
| 283.3233 @ 1.3100004 | 283.3233 | 1.3100004 | 0.532 | 0.015 | | 0.016 |
| 262.9351 @ 4.2200007 | 262.9351 | 4.2200007 | -0.449 | 0.311 | | 0.047 |
| C31 H30 N2 O7 S2 | 606.1501 | 6.9110045 | 0.541 | 0.257 | | 0.014 |
